# Comparing Neurodevelopmental Outcomes in Infants With Patent Ductus Arteriosus Stenting Versus Blalock-Taussig-Thomas Shunt: A Pilot Study

**DOI:** 10.1016/j.jscai.2024.101355

**Published:** 2024-03-04

**Authors:** Howaida El-Said, Amira Hussein, Katherine Price, Jessica Heibel, Jessica Haley, Shylah Haldeman, Zeinab Boulil, Matthew Brigger, Aparna Rao, Srujan Ganta, Rohit Rao, John Nigro, Nathaly Sweeney

**Affiliations:** a Department of Pediatrics, University of California San Diego, La Jolla, California; b Division of Cardiology, Rady Children’s Hospital-San Diego, San Diego, California; c University of California San Diego School of Medicine, La Jolla, California; d Department of Otolaryngology, University of California San Diego, La Jolla, California; e Division of Pediatric Otolaryngology, Rady Children’s Hospital-San Diego, San Diego, California; f Division of Pediatric Pulmonology, Rady Children’s Hospital-San Diego, San Diego, California; g Department of Surgery, University of California San Diego, La Jolla, California; h Division of Cardiothoracic Surgery, Rady Children’s Hospital-San Diego, San Diego, California; i Division of Neonatology, Rady Children’s Hospital-San Diego, San Diego, California

**Keywords:** Blalock-Taussig-Thomas shunt, congenital heart disease, feeding skill acquisition, gastrostomy tube, neurodevelopment, patent ductus arteriosus stent

## Abstract

**Background:**

Patent ductus arteriosus stenting (PDAS) is a nonsurgical alternative to Blalock-Taussig-Thomas shunt (BTTS) for infants with ductal-dependent congenital heart disease. In this single-center study, we aimed to compare neurodevelopmental outcomes in children who underwent BTTS as initial palliation versus PDAS.

**Methods:**

Bayley Scales of Infant and Toddler Development Screening Test (Bayley-III) reports and mode of feeding data were collected for any patient who underwent PDAS or BTTS at Rady Children’s Hospital from 2013 to 2021. We also prospectively administered the Parents’ Evaluation of Development Status questionnaire (PEDS) to parents of children aged 2-8 years in this patient population.

**Results:**

Of the 99 patients, 64 received a Bayley-III assessment and/or PEDS screen. Of the 35 who had a Bayley-III, there was a higher proportion of patients with PDAS who scored as developmentally appropriate compared with BTTS. PEDS screen showed that a higher proportion of patients with PDAS had no parental concern for delay than that of patients with BTTS (63% vs 30%). Patients with BTTS were more likely to undergo gastrostomy tube placement than patients with PDAS.

**Conclusions:**

Our study suggests that neurodevelopmental measures are feasible, clinically relevant, and should be included in comparative effectiveness studies of infant congenital interventions. Whether PDAS offers neurodevelopmental benefit over BTTS should be confirmed in a prospective powered randomized controlled clinical trial.

## Introduction

Children with congenital heart disease (CHD) are at increased risk of neurodevelopmental morbidity not only due to the inherent physiology of their cardiac disease but also secondary to underlying biological processes such as genetic disorders. Furthermore, medical and surgical advancements that have been critical in increasing survival in children with CHD are also associated with increased negative impact on neurodevelopmental outcomes.^1^, ^2^, ^3^, ^4^ With drastically improved survival of patients with CHD over time, there has been an increasing focus on optimizing long-term outcomes and neurodevelopment in these children.^5^ Relative neurodevelopmental deficits have been shown in nearly all domains, including gross and fine motor skills, language and social skills, executive functioning, problem solving, and memory in survivors of CHD.^2^^,^^6^^,^^7^ The emergence of percutaneous patent ductus arteriosus stenting (PDAS) as a noninferior or possibly superior alternative to Blalock-Thomas-Taussig shunt (BTTS) placement for initial palliation in infants with ductal-dependent CHD^8^, ^9^, ^10^, ^11^, ^12^ presents a unique opportunity to mitigate surgery-related risk factors for neurodevelopmental impairment by delaying surgical interventions until later in infancy/childhood. Since 2017, our institution has adopted PDAS as the standard of care for most ductal-dependent CHD given these perceived advantages. In this single-center study, we compare neurodevelopmental outcomes in children who underwent BTTS placement with those in children who underwent PDAS.

## Methods

We performed a retrospective chart review for any patient who underwent BTT shunt placement or PDA stenting from 2013-2021 at Rady Children’s Hospital in San Diego, California, and collected Bayley Scales of Infant and Toddler Development Screening Test, Third Edition (Bayley-III) reports for both groups. Only patients who had received a complete Bayley-III assessment during the above time period were included. To screen for the most recent neurodevelopmental status, the Parents’ Evaluation of Developmental Status (PEDS) questionnaire was administered to parents of children ages 2 to 8 years from the above- mentioned cohort who had previously undergone BTTS placement or PDAS. Mode of feeding data for all patients were collected through retrospective chart review and comparisons were made between the BTTS and PDAS groups at different timepoints peri cardiac interventions. The corresponding author had full access to all the data in the study and takes responsibility for its integrity and the data analysis. This study was approved by the University of California Institutional Review Board (#201338).

## Results

Of the 99 eligible patients who underwent BTTS placement or PDAS from 2013 to 2021, 64 received a complete Bayley-III Assessment and/or PEDS questionnaire screening. No difference was found between the demographics of those patients who received a neurodevelopmental assessment and those who did not (Supplemental Table S1). In addition, the sampled cohort from each intervention group, PDAS or BTTS, were similar to the available patients for each intervention group in the original cohort (Table 1). This shows that the sampled patients were a good representation of the entire cohort.

**Table 1:** Patient demographics comparing sampled cohort with entire cohort stratified by PDAS and BTTS.

Demographics	Entire cohort of patients 2012-2021		Sampled cohort of patients	
	PDAS (n = 58)	BTTS (n = 41)	PDAS (n = 35)	BTTS (n = 29)
Female sex	28 (48)	12 (29)	16 (46)	10 (34)
Race/ethnicity				
White	26 (45)	17 (42)	16 (45)	13 (45)
Black/African American	5 (9)	2 (5)	1 (3)	1 (3)
Asian	2 (3)	3 (7)	2 (6)	2 (7)
Native Hawaiian/other Pacific Islander	3 (5)	5 (12)	1 (3)	3 (10)
Other	22 (38)	14 (34)	15 (43)	10 (35)
Hispanic or Latino	29 (50)	18 (44)	19 (54)	15 (52)
Single vs biventricular repair candidate				
Biventricular	23 (40)	23 (56)	16 (46)	16 (55)
Single ventricle	20 (34)	12 (29)	11 (31)	10 (34)
Other	15 (26)	6 (15)	8 (23)	3 (10)
Syndrome				
Yes	13^c^ (22)	2^d^ (5)	13^e^ (37)	2^f^ (7)
No	45 (78)	39 (95)	22 (63)	27 (93)
Cardiac diagnosis^a^				
TOF	7 (12)	20 (49)	3 (9)	12 (41)
PA/IVS	21 (36)	4 (10)	12 (34)	4 (14)
Single ventricle PA/PS	13 (22)	9 (22)	8 (23)	7 (24)
D-TGA/PS	4 (1)	3 (7)	3 (9)	2 (7)
Heterotaxy/AV canal/PS	5 (8)	0 (0)	5 (14)	0 (0)
PA/VSD	6 (10)	4 (10)	1 (3)	3 (10)
Severe PS	6 (10)	1 (2)	3 (9)	1 (3)
Ebstein’s anomaly	3 (5)	1 (2)	1 (3)	1 (3)
LdCAVC	1 (2)	0 (0)	1 (3)	0 (0)
Other	24 (40)	2 (5)	12 (34)	1 (3)
Mode of feeding at time of shunt or stent				
Exclusive PO	22 (38)	14 (34)	10 (29)	12 (41)
Not exclusive PO^b^	36 (62)	27 (66)	25 (71)	17 (59)
Mode of feeding at time of Glenn or definitive surgery				
Exclusive PO	31 (53)	22 (54)	18 (51)	14 (48)
Not exclusive PO^b^	9 (16)	15 (36)	7 (20)	13 (45)
Unknown	18 (31)	4 (10)	10 (29)	2 (7)
Mode of feeding at most recent follow-up				
Exclusive PO	46 (79)	28 (68)	29 (83)	22 (76)
Not exclusive PO^b^	9 (16)	7 (17)	6 (17)	5 (17)
Unknown	3 (5)	6 (15)	0 (0)	2 (7)
Gastrostomy tube feedings				
Yes	12 (21)	19 (46)	8 (23)	15 (52)
No	43 (74)	22 (54)	26 (74)	14 (48)
Unknown	3 (5)	0 (0)	1 (3)	0 (0)

Values are n (%).

AV, atrioventricular; BTTS, Blalock-Taussig-Thomas shunt; DILV, double inlet left ventricle; D-TGA, dextrotransposition of the great arteries; LdCAVC, left dominant complete atrioventricular canal; PA, pulmonary atresia; PA/IVS, pulmonary atresia/intact ventricular septum; PDAS, patent ductus arteriosus stenting; PO, oral intake; PS, pulmonary stenosis; TOF, tetralogy of Fallot; VSD, ventricular septal defect.

a Some patients had more than 1 diagnosis.

b See Supplemental Table S6 for detailed mode of feeding description for the whole cohort (n = 99).

c Trisomy 21 (n = 2), 22q11.2 (n = 4), heterotaxy (n = 6), other (n = 3).

d Trisomy 21 (n = 2), 22q11.2 (n = 3), heterotaxy (n = 6), other (n = 4).

e Trisomy 21 (n = 1), 22q11.2 (n = 2), heterotaxy (n = 6), other (n = 4).

f 22q11.2 (n = 1), trisomy 21 (n = 1).

### Bayley-III neurodevelopmental assessments

**Figure 1:**
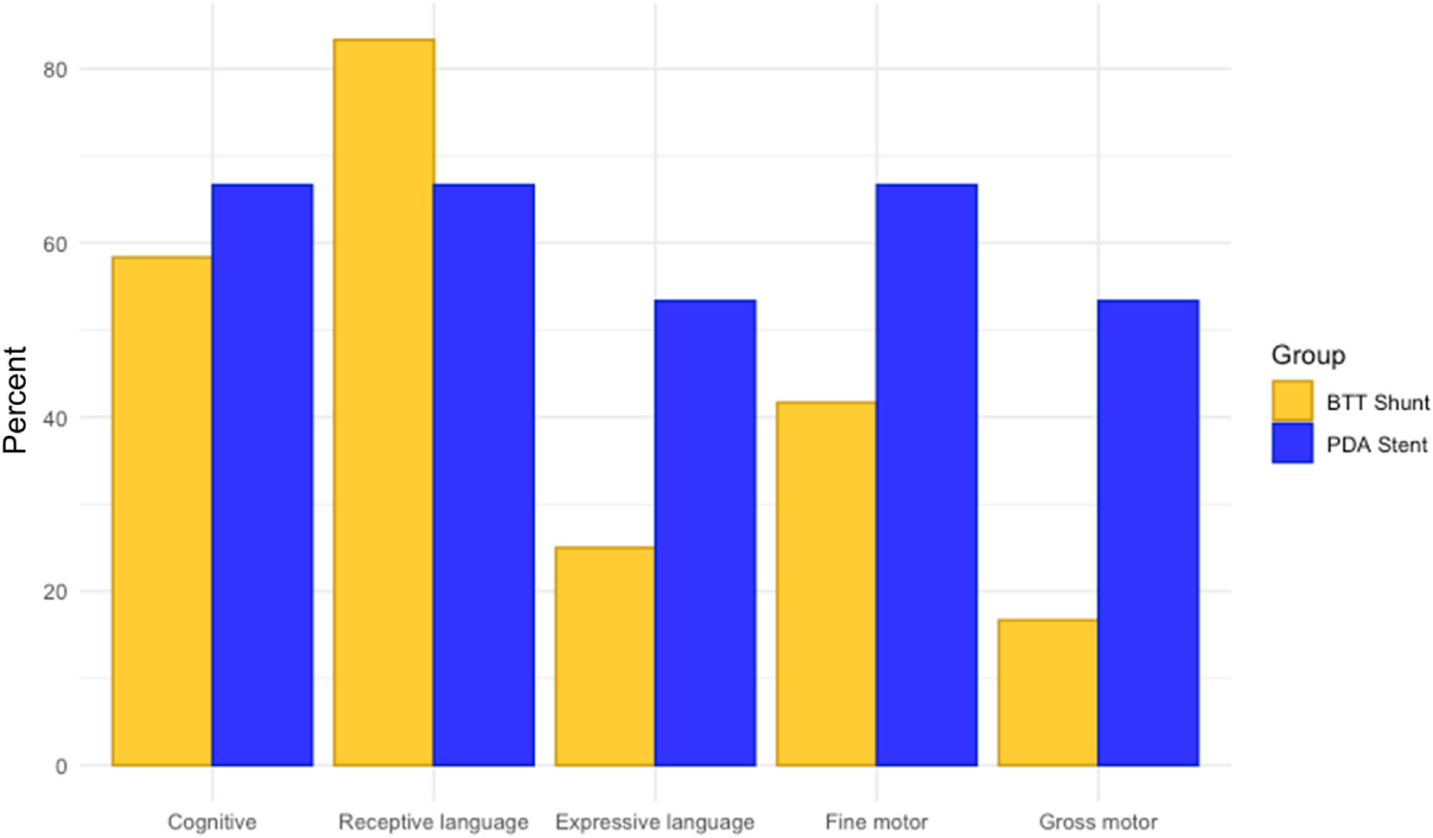
**Percentages of patients aged 12 months or younger who scored developmentally appropriate on Bayley-III Assessment.** BTTS, Blalock-Taussig-Thomas shunt; PDA, patent ductus arteriosus.

All patients with structural CHD treated at our institution are referred to our High-risk Infant Follow-up clinic for periodic neurodevelopmental assessment. Thirty-five (35%) patients had a complete Bayley-III assessment at 1 time point during their medical care, with 16 (46%) in the BTTS group and 19 (54%) in the PDAS group. The rate of attrition for follow-up with High-risk Infant Follow-up clinic was rather high after 12 months of age. Although 27% (27) of the cohort had a complete Bayley-III assessment by 12 months of age, only 15% (16) had a complete Bayley-III assessment after 12 months of age. Analysis of the available data for the whole cohort showed that a higher proportion of patients in the PDAS group scored developmentally appropriate in the cognitive, expressive language, fine motor, and gross motor skills domains. (Supplemental Table S2). The same pattern was observed when the analysis was repeated excluding patients with known genetic syndromes that can impact neurodevelopment (Supplemental Table S3). To assess for potential areas that could benefit from early intervention, we reran the analysis but this time limiting to the group with complete Bayley-III assessment by 12 months of age, which was also the largest number of complete Bayley-III assessments. Of the 27 patients who had complete Bayley-III assessments by the age of 12 months, 12 (44%) underwent BTTS placement and 15 (56%) PDAS. There was a trend toward better performance in cognitive, expressive language, fine and gross motor skills in the PDAS group, but a slightly higher proportion of the patients in the BTTS group performed better in the receptive language domain (Table 2, Figure 1). A similar performance pattern was observed when patients with genetic syndromes known to affect neurodevelopment (Trisomy 21 and 22q11.2 deletion syndrome) were excluded from the analysis (Supplemental Table S4 and Supplemental Figure S1).

**Table 2:** Bayley-III assessments for all patients aged 12 months or younger.

Bayley-III at age 12 mo or younger	BTTS (n = 12)		PDAS (n = 15)	
	Developmentally appropriate patients	Developmentally delayed patients	Developmentally appropriate patients	Developmentally delayed patients
Cognitive	7 (58)	5 (42)	10 (67)	5 (33)
Receptive language	10 (83)	2 (17)	10 (67)	5 (33)
Expressive language	3 (25)	9 (75)	8 (53)	7 (47)
Fine motor	5 (42)	7 (58)	10 (67)	5 (33)
Gross motor	2 (17)	10 (83)	8 (53)	7 (47)

Values are n (%).

PDA, patent ductus arteriosus.

### PEDS questionnaire

**Figure 2:**
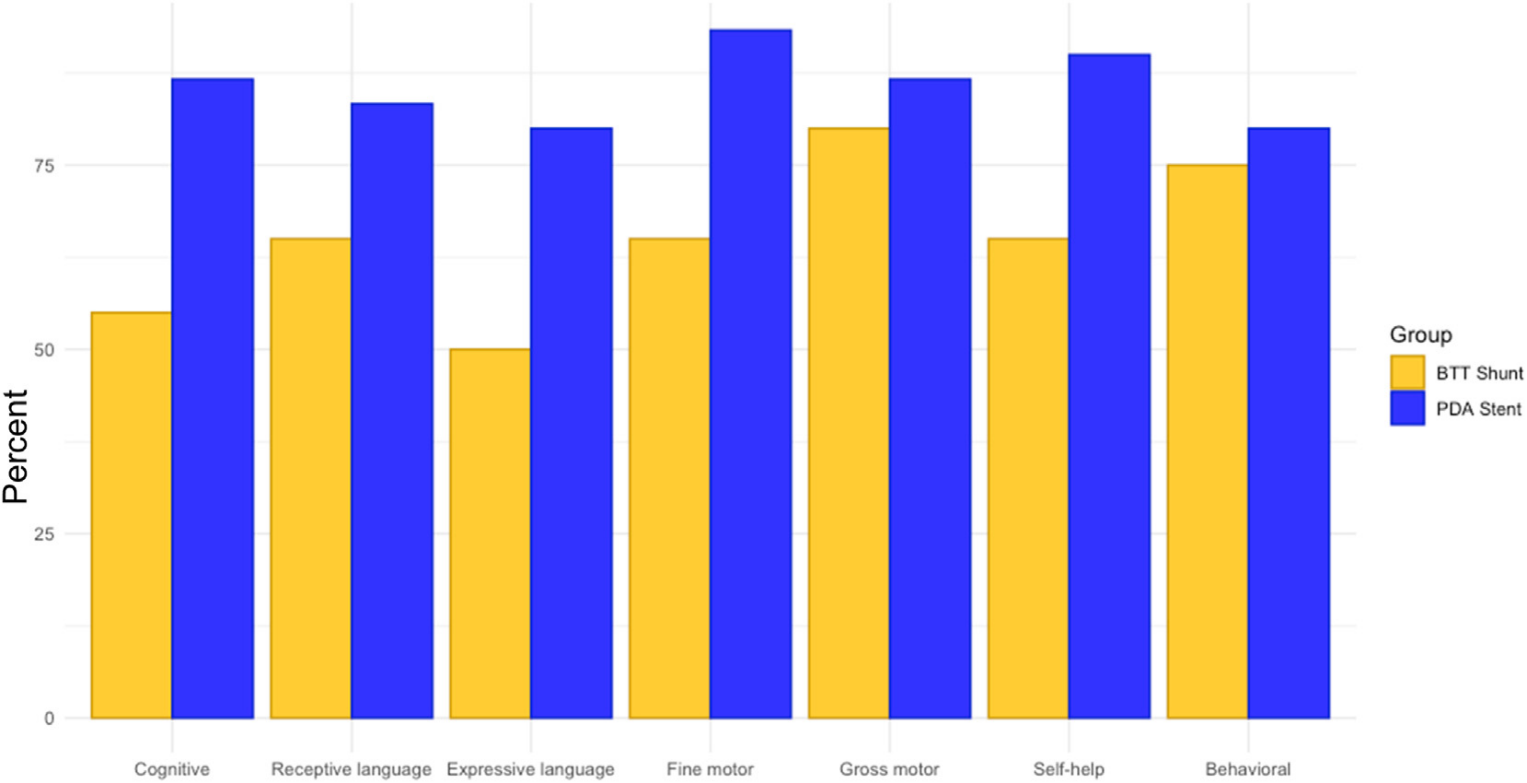
**Percentages of low-risk category patients (no parental concern for developmental delay) on PEDS questionnaire for all patients ages 2 to 8 years.** Detailed information on age range at time of PEDS questionnaire are available in Supplemental Tables S8 and S9. BTTS, Blalock-Taussig-Thomas shunt; PDA, patent ductus arteriosus; PEDS, Parents’ Evaluation of Development Status.

All eligible parents in the original cohort were approached by researchers for the administration of the PEDS questionnaire. The use of caregiver questionnaire in addition to direct child testing is supported by the cardiac neurodevelopmental outcome collaborative as part of the Core Neurodevelopmental Assessment Battery.^13^ The PEDS questionnaire was selected for its ease of administration to a wide age range (ages 0-8) to encourage engagement and screen as many children in the original cohort as possible with the same instrument. Fifty parents completed the questionnaire for their children, 20 (40%) had previously undergone BTTS placement and 30 (60%) PDAS. The percentage of patients who completed the PEDS questionnaire closely resembles the percentage of patients available from each group in the original cohort, 41% of the original cohort were in the BTTS group and 59% in the PDAS group. We found that the patients in the PDAS group had lower proportion of patients with moderate/high concern for developmental delay than patients in the BTTS group (37% PDAS, 70% BTTS) (Table 3, Figure 2). Furthermore, when looking at the specific areas of moderate/high concern, there were higher percentages of patients in the BTTS group with concerns in the cognitive, expressive language, and fine motor domains. The PDAS group had a higher proportion of patients score in the low-risk category (no concern for delay) in the receptive language, gross motor, self-help, and behavioral domains than the BTTS group (Table 3, Figure 2).

**Table 3:** Areas of parental concerns as evaluated by the PEDS questionnaire

PEDS at ages 2-8 y^a^	PDAS (n = 30)		BTTS (n = 20)	
	No concern for delay (low-risk category)	Concern for delay (moderate or high-risk category)	No concern for delay (low-risk category)	Concern for delay (moderate-risk or high-risk category)
Overall	19 (63)	11 (37)	6 (30)	14 (70)
Cognitive	26 (87)	4 (13)	11 (55)	9 (45)
Receptive language	25 (83)	5 (17)	13 (65)	7 (35)
Expressive language	24 (80)	6 (20)	10 (50)	10 (50)
Fine motor	28 (93)	2 (7)	13 (65)	7 (35)
Gross motor	26 (87)	4 (13)	16 (80)	4 (20)
Self-help	27 (90)	3 (10)	13 (65)	7 (35)
Behavioral	24 (80)	6 (20)	15 (75)	5 (25)

Values are n (%).

BTTS, Blalock-Taussig-Thomas shunt; PDAS, patent ductus arteriosus stent.

a Detailed information on age range at time of PEDS questionnaire are available in Supplemental Tables S8 and S9.

When patients with genetic syndromes known to affect neurodevelopment were excluded from the analysis, the BTTS group continued to have a higher proportion of patients with concerns for delay in the cognitive and expressive language domains than the PDAS group, but not in the fine motor domain. A similar trend was seen in the receptive language domain (Supplemental Table S5, Supplemental Figure S2). It should be noted that because a larger proportion of the children included in the BTTS group were older at the time of PEDS assessment (average age BTTS group vs PDAS group: 6.2 vs 4.8 [Table 3] and 5.6 vs 4.7 [Supplemental Table S5], respectively), potentially, some children in the PDAS group may go on to exhibit more developmental concerns as they age.

### Acquisition of feeding skills

CHD is associated with significant barriers in achieving full oral feeding. Factors such as mechanical ventilation and surgical intervention for palliation of CHD further complicates the process because they interfere with acquisition of these skills during a critical time of development.^14^ To ascertain the effect of PDAS and BTTS placement on enteral feeding, we collected data on the mode of feeding for all patients in the original cohort. From the 64 patients who received a Bayley-III Assessment and/or PEDS questionnaire, 29 (45%) underwent BTTS placement and 35 (55%) PDAS. We evaluated mode of feeding at 3 time points: first, at the time of BTTS or PDAS placement; second, at the time of Glenn or definitive surgery; and, finally, at the time of most recent follow-up. We found that at the time of PDAS or BTTS, the proportion of infants feeding orally was similar in both groups in the original cohort. The proportion of infants feeding orally in the sampled cohort was slightly higher for the BTTS group. At the time of Glenn or definitive surgery, a higher proportion of the patients in the BTTS group in the original cohort and sampled cohort required oral feeding supplementation in the form of nasoenteric or gastrostomy tube (GT) feeds compared with that of the patients in the PDAS group (Table 4, Supplemental Table S6). The proportion of patients for whom there was no feeding mode information available at the time of Glenn or definitive surgery was also higher in the sampled PDAS group. Feeding mode at the most recent follow-up was similar across the groups.

**Table 4:** Mode of feeding for all patients who received a Bayley-III Assessment and/or PEDS questionnaire

	PDAS (n = 35)	BTTS (n = 29)
Mode of feeding at time of PDAS or BTTS		
Exclusive PO	10 (29)	12 (41)
Not exclusive PO^a^	25 (71)	17 (59)
Mode of feeding at time of Glenn or definitive surgery		
Exclusive PO	18 (51)	14 (48)
Not exclusive PO^a^	7 (20)	13 (45)
Unknown	10 (29)	2 (7)
Mode of feeding at the most recent follow-up		
Exclusive PO	29 (83)	22 (76)
Not exclusive PO^a^	6 (17)	5 (17)
Unknown		2 (7)
Gastrostomy tube feedings		
Yes	8 (23)	15 (52)
No	26 (74)	14 (48)
Unknown	1 (3)	0 (0)

Values are n (%).

BTTS, Blalock-Taussig-Thomas shunt; PDAS, patent ductus arteriosus stent; PO, oral intake.

a Detailed mode of feeding for each time point can be found in Supplemental Table S6.

Evaluation of the prevalence of GTs showed that the patients in the BTTS group were more likely to have received a GT for feeding support (Table 4, Supplemental Table S6). At our institution, GT placement is only pursued if the child fails to/is unable to attain the skills necessary to take oral feeds and fails a period of nasogastric/jejunal tube feedings as outpatient. It is our practice to discharge patients on nasogastric tube feedings with outpatient follow-up to allow more time for feeding skill acquisition. It could be inferred that because a larger proportion of the children included in the BTTS group were older, potentially, some children in the PDAS group may also go on to require GT placement later. This assumption would be rather unlikely given the fact that only 1 child in the PDAS group was still receiving supplemental nutrition in the form of total parenteral nutrition at the time of the most recent follow-up. The rest were all fully oral fed, oral/GT fed (3), or fully GT fed (5).

When the analysis was repeated with exclusion of patients with genetic syndromes known to affect neurodevelopment, there was a higher number of patients in the BTTS group who were exclusively oral feeding at the time of first intervention. When looking at exclusive oral feeding at the time of definitive surgery/Glenn and most recent follow-up, there was a trend toward more patients in the PDAS group achieving exclusive oral feeding. The proportion of patients receiving supplemental enteral feedings was higher in the BTTS group at the time of Glenn or definitive surgery. The prevalence of GT placement remained higher in the BTTS group (Supplemental Table S7).

## Discussion

Although surgical and interventional advances have led to the improved survival of children with CHD, there are concerns about negative effects on neurodevelopmental outcomes. Significant effort is being placed in mitigating the effect of these interventions and maximize positive neurodevelopment in these children. Our data suggest that the use of PDAS as the initial palliative procedure may be associated with improved performance on the Bayley-III assessment, although only nominally (Central Illustration). These findings may be due to the small sample size of our study. Furthermore, parents of children who underwent BTTS placement for initial palliation were more likely to report moderate to high neurodevelopmental concerns than parents of children who underwent PDAS. Factors associated with poor neurodevelopmental outcomes linked to surgical interventions are numerous. Surgical interventions are inherently associated with prolonged sedation, mechanical ventilation, prolonged hospitalization, and multiple vascular access for monitoring, which increase the risk of infection and further prolong hospitalization. Furthermore, patients with CHD are frequently at risk for poor neurodevelopmental outcomes owing to unavoidable factors inherent to their cardiac defect that may limit brain oxygenation, cause in utero circulatory abnormalities that may impair cerebral autoregulation, and lead to abnormal neonatal transition physiology.^15^

**Central Illustration:**
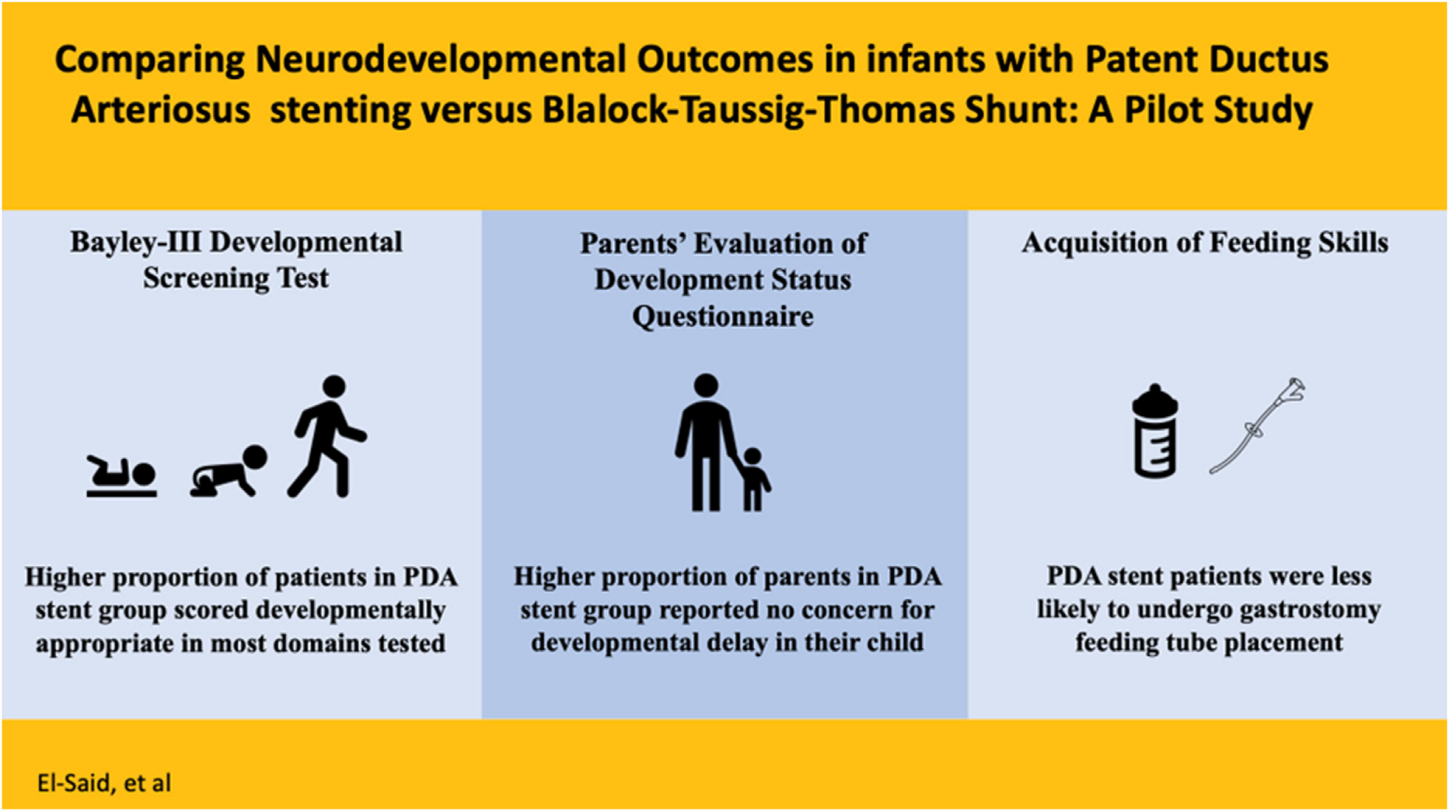
A higher proportion of patients in the PDAS group scored developmentally appropriate in the cognitive, expressive language, fine motor, and gross motor skills domains. Patients in the PDAS group had lower proportion of patients with moderate/high parental concern for developmental delay than patients in the BTTS group (37% PDAS, 70% BTTS). Patients in the BTTS group were more likely to have received a gastrostomy tube for feeding support. BTTS, Blalock-Taussig-Thomas shunt; PDAS, patent ductus arteriosus stenting.

In this small sample–sized cohort, there is suggestion that there may be better outcomes in the PDAS group in most areas compared with those in the BTTS group. Based on these findings, we propose that children who avoid palliative surgical shunt in infancy may be at lower risk of poor neurodevelopmental outcomes. The factors contributing to the increased risk of neurodevelopmental delays in patients with CHD are myriad. Besides the likely unavoidable factors inherent to CHD as described earlier, additional factors such as exposure to multiple medical and surgical interventions on already vulnerable brain tissue, insufficient growth and nutrition due to increased metabolic demand, medical comorbidities, and parental stress and anxiety further impact neurodevelopmental outcomes in these patients.^3^^,^^16^^,^^17^ Surgical risk factors implicated in poor neurodevelopmental outcomes such as deep hypothermic circulatory arrest, cardiopulmonary bypass, end-organ reperfusion injury, intraoperative acid-base imbalances, anesthesia, and analgesia have been well described.^3^^,^^16^^,^^17^ Furthermore, surgical interventions are frequently associated with postoperative low cardiac output and hypotension, which may impair brain perfusion and postoperative pain and agitation that further increase brain metabolic demand. Surgical interventions also lead to prolonged hospitalization and increased intensive care unit length of stay and frequently place the infant in a state that limits their ability to interact with caregivers and their environment, all factors associated with negative impact on neurodevelopment.^3^^,^^16^^,^^17^ The impact of surgical interventions may be mitigated by avoidance of the operating room whenever possible.

Another area of neurodevelopment frequently compromised in the infant with CHD is nutrition. Children with CHD frequently have delayed feeding initiation and abnormal acquisition of feeding skills in infancy. These children are frequently separated from their mother soon after delivery; some require preoperative mechanical ventilation and/or have tenuous preoperative end-organ perfusion impairing adequate nutrition. Furthermore, postoperative hypermetabolic state and prolonged intubation period can exacerbate the already vulnerable energy imbalance in these patients.^18^^,^^19^ Inadequate nutrition impairs normal physical growth and can, in turn, negatively affect neurodevelopment. It has also been associated with poor long-term cognitive and motor outcomes.^18^^,^^20^, ^21^, ^22^, ^23^ This is particularly true for children dependent on enteric tube feeds^24^ and with genetic syndromes.^18^ In our cohort, a higher proportion of infants in the BTTS group received nasoenteric or GT feeding supplementation compared with that of those in the PDAS group at the time of Glenn or definitive surgery. Furthermore, the children who underwent BTTS placement were more likely to receive surgical GT placement for feeding. These findings may suggest increased rates of feeding difficulties in the BTTS group, which potentially resulted from impaired feeding skills acquisition and further impacted neurodevelopment of these patients. Similarly, in a previous study, although no difference was detected in interstage growth between PDAS and BTTS groups, the authors found that those who underwent PDAS had a simpler feeding regimen and fewer feeding-related readmissions.^25^

To our knowledge, this is the first study comparing neurodevelopmental outcomes of children who underwent BTTS placement with those who underwent PDAS and potentially adds to the growing body of data supporting the PDAS as initial palliation versus BTTS placement. Multiple meta-analyses have demonstrated that compared with BTTS, PDAS confers several significant advantages in the population of infants with ductal-dependent CHD. Fakhry et al^9^ found that compared with BTTS, PDAS resulted in reduced risk of mortality, procedural complications, and extracorporeal membrane oxygenation and higher postprocedural oxygen saturation and Nakata index. Similarly, Alsagheir et al^8^ found that compared with BTTS, PDAS was associated with a lower risk of procedural complications and medium-term mortality, although the PDAS group required a higher rate of reintervention. In addition, the meta-analysis by Li et al^10^ demonstrated that the stent group had lower mortality and complication rates and a shorter hospital and intensive care unit length of stay, no difference was found in pulmonary artery growth or unplanned reintervention rates. Bauser-Heaton et al^11^ also found lower mortality rates and a shorter hospital length of stay in the stent group, although at the expense of increased reintervention rates.

### Limitations

Several of the strengths of this study are also its limitations. As a single-center study, it allows us to control for variations in patient triage and management, including referral process for PDAS versus BTTS placement, preinterventional/postinterventional management and feeding practices, but also limits generalization. Moreover, because our institution implemented PDAS as the default initial palliative strategy for all suitable patients with ductal-dependent CHD in 2017 but before that regarded BTTS as the standard of care, there may exist an era effect that may impact outcomes including feeding practices. Moreover, the number of complete Bayley-III assessments available for our total patient population was limited; because this assessment requires a relatively extensive in-person evaluation by trained professionals, the families that presented for this assessment may differ in socioeconomic status, baseline neurodevelopmental status, parental support, and patient’s health status. The PEDS questionnaire was administered through telephone, which mitigates some of these factors, but the outcome measures of this questionnaire are solely parent reported and, therefore, prone to bias. Finally, the patients who received a BTTS versus a PDAS may differ in baseline anatomical risk, which could further impact neurodevelopmental outcomes. This phenomenon was suggested by Bauser-Heaton et al,^11^ who found that patients who received a BTTS were more likely to have single-ventricle anatomy or single-source pulmonary blood flow. However, in our cohort, the proportion of patients with single-ventricle anatomy were equivalent in the BTTS and PDAS groups in both the original and sampled cohorts. We did see differences in cardiac diagnosis in the original and sampled cohorts where there were more patients with tetralogy of Fallot in the BTTS group than those in the PDAS group. In addition, the BTTS group had less patients with a diagnosis of pulmonary atresia/intact ventricular septum and no patients with heterotaxy syndrome. Finally, the sample size of the cohorts did not meet sufficient statistical power, limiting this study to a descriptive analysis.

## Conclusions

Our study suggests that neurodevelopmental measures are feasible, are clinically relevant, and should be included in comparative effectiveness studies of infant congenital interventions. Whether PDAS offers neurodevelopmental benefit over BTTS should be confirmed in a prospective powered randomized controlled clinical trial.
