# Supplementary material for: Comparing Neurodevelopmental Outcomes in Infants With Patent Ductus Arteriosus Stenting Versus Blalock-Taussig-Thomas Shunt: A Pilot Study
Source: J Soc Cardiovasc Angiogr Interv. 2024 Mar 4;3(5):101355. doi: 10.1016/j.jscai.2024.101355 (PMC11307534; doi:10.1016/j.jscai.2024.101355)
Supplement: Supplementary Material [file mmc1.docx]

**Supplemental Material**

**Supplemental Table S1. Patient Demographics Entire Cohort versus Sampled Cohort. There was no significant difference in the demographics of the entire cohort of patients who received a BTTS or PDAS from 2013-2021 and the subset of patients who received a neurodevelopmental assessment as part of this study. TOF: Tetralogy of Fallot. PA/IVS: pulmonary atresia/intact ventricular septum. PA: pulmonary atresia. PS: pulmonary stenosis. D-TGA: dextro-transposition of the great arteries. AV: atrioventricular. VSD: ventricular septal defect. DILV: double inlet left ventricle. LdCAVC: left dominant complete atrioventricular canal. ^a^Some patients have more than one diagnosis. ^b^See Supplemental Table S3 for detailed mode of feeding description for the whole cohort (n=99). ^c^Trisomy 21(2), 22q11.2(4), Heterotaxy(6), Other (3). ^d^Trisomy 21(2), 22q11.2 (3), Heterotaxy (6), Other (4).**

| **Demographics** | | **Entire cohort of patients who underwent BTTS or PDAS (n=99)** | **Subset of patients who received Bayley-III Assessment and/or PEDS Questionnaire (n=64)** |
| --- | --- | --- | --- |
| Sex | Male  Female | 59 (60%)  40 (40%) | 38 (60%)  26 (40%) |
| Race | White  Black/African American  Asian  Native Hawaiian/Other Pacific Islander  Other | 43 (43%)  7 (7%)  5 (5%)  8 (8%)  36 (36%) | 29 (45%)  2 (3%)  4 (6%)  4 (6%)  25 (39%) |
| Ethnicity | Hispanic or Latino  Not Hispanic or Latino | 47 (47%)  52 (53%) | 34 (53%)  30 (47%) |
| Single vs. Biventricular Repair Candidate | Biventricular  Single Ventricle  Other | 46 (47%)  32 (32%)  21 (21%) | 32 (50%  21 (33%)  11 (17%) |
| Syndrome | Yes  No | 15^c^(15%)  84 (85%) | 15^d^ (23%)    49 (77%) |
| Cardiac Diagnosis^a^ | TOF  PA/IVS  Single Ventricle PA/PS  D-TGA/PS  Heterotaxy/AV Canal/PS  PA/VSD  Severe PS  Ebstein’s Anomaly  LdCAVC  Other | 27 (27%)  25 (25%)  22 (22%)  7 (7%)  5 (5%)  10 (10%)  7 (7%)  4 (4%)  1 (1%)  26 (26%) | 15 (23%)  16 (25%)  15 (23%)  5 (8%)  5 (8%)  4 (6%)  4 (6%)  2 (3%)  1 (2%)  13 (20%) |
| Mode of Feeding at Time of Shunt or Stent | Exclusive PO  Not exclusive PO^b^ | 36 (36%)  63 (64%) | 22 (34%)  42 (66%) |
| Mode of Feeding at Time of Glenn or Definitive Surgery | Exclusive PO  Not exclusive PO^b^  Unknown | 53 (54%)  24 (24%)  21 (21%) | 32 (50%)  20 (31%)  12 (19%) |
| Mode of Feeding at Most Recent Follow-up | Exclusive PO  Not exclusive PO^b^  Unknown | 74 (75%)  16 (16%)  9 (9%) | 51 (80%)  11 (17%)  2 (3%) |
| Gastrostomy Tube Feedings | Yes  No  Unknown | 31 (31%)  65 (66%)  3 (3%) | 23 (36%)  40 (63%)  1 (1%) |

**Supplemental Table S2. Available Complete Bayley-III Assessment for original cohort**

**There was a higher proportion of patients in the PDAS group compared to the BTT group who tested developmentally age-appropriate in all areas tested except receptive language, although this did not reach statistical significance. DA= Developmentally Appropriate. DD= Developmentally Delayed.**

| All complete Bayley-III assessments | | | | | | |
| --- | --- | --- | --- | --- | --- | --- |
|  | **Surgical** | | **Total** | **Stent** | | **Total** |
|  | **DA** | **DD** | **16** | **DA** | **DD** | **19** |
| Cognitive | **10** | **6** | **62.50%** | **13** | **6** | **68.40%** |
| Receptive Language | **11** | **5** | **68.75%** | **11** | **8** | **57.89%** |
| Expressive Language | **4** | **12** | **25%** | **9** | **10** | **47.37%** |
| Fine Motor | **7** | **9** | **43.75%** | **11** | **8** | **57.89%** |
| Gross Motor | **4** | **12** | **25%** | **8** | **11** | **42%** |

**Supplemental Table S3. Available Complete Bayley-III Assessment for original cohort minus known genetic syndromes. There was a higher proportion of patients in the PDAS group compared to the BTT group who tested developmentally age-appropriate in all areas tested except receptive language, although this did not reach statistical significance. DA= Developmentally Appropriate. DD= Developmentally Delayed.**

| All complete Bayley-III assessments  Minus known Genetic Syndromes | | | | | | |
| --- | --- | --- | --- | --- | --- | --- |
|  | **Surgical** |  | **Total** | **Stent** |  | **Total** |
|  | **DA** | **DD** | **14** | **DA** | **DD** | **18** |
| Cognitive | **9** | **5** | **64.30%** | **13** | **5** | **72.22%** |
| Receptive Language | **9** | **5** | **64.30%** | **11** | **7** | **61.11%** |
| Expressive Language | **4** | **10** | **28.57%** | **9** | **9** | **50%** |
| Fine Motor | **6** | **8** | **42.86%** | **11** | **7** | **61.11%** |
| Gross Motor | **4** | **10** | **28.57%** | **8** | **10** | **44.44%** |

**Supplemental Table S4. Bayley-III Assessment results for patients ≤ 12 months of age, excluding those with genetic syndromes known to affect neurodevelopment.** Patients in the PDA stent group performed better than the BTT shunt group in all areas except receptive language.

| **Bayley-III at age ≤ 12 months** | **BTT Shunt (n=11)** | | **PDA Stent (n=14)** | |
| --- | --- | --- | --- | --- |
|  | **Number of Developmentally Appropriate Patients** | **Number of Developmentally Delayed Patients** | **Number of Developmentally Appropriate Patients** | **Number of Developmentally Delayed Patients** |
| **Cognitive** | 6 (55%) | 5 (45%) | 10 (71%) | 4 (29%) |
| **Receptive Language** | 9 (82%) | 2 (18%) | 10 (71%) | 4 (29%) |
| **Expressive Language** | 3 (27%) | 8 (73%) | 8 (57%) | 6 (43%) |
| **Fine Motor** | 4 (36%) | 7 (64%) | 10 (71%) | 4 (29%) |
| **Gross Motor** | 2 (18%) | 9 (82%) | 8 (57%) | 6 (43%) |

**Supplemental Figure S1. Percentages of patients ≤ 12 months of age who scored developmentally appropriate on Bayley-III Assessment, excluding those with genetic syndromes known to affect neurodevelopment. This shows a general trend toward higher proportions of developmentally appropriate patients in the PDA stent group compared to the BTT shunt group in all areas except**

**receptive language.**


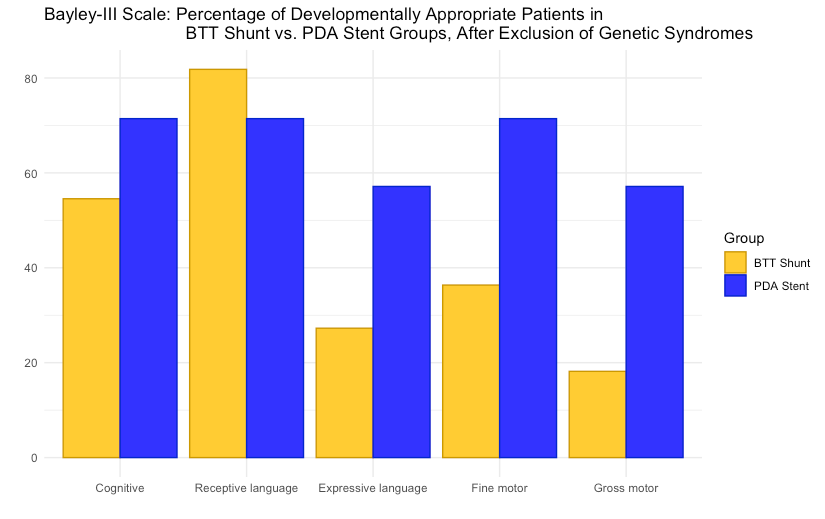


**Supplemental Table S5. Areas of parental concerns as evaluated by the PEDS Questionnaire, excluding children with genetic syndromes known to affect neurodevelopment. The PDA stent group had higher percentages of patients score in the low-risk category (no concern for delay) in the cognitive and expressive language domains compared to the BTT shunt group. A similar trend was seen in the receptive language and self-help domains, Average age in the BTTS group at the time of PEDS questionnaire was ~5.6 while it was ~4.7 in the PDAS group. ^a^Detailed information on age range at time of PEDS questionnaire are available in Supplemental Tables S8 and S9.**

| **PEDS at ages 2-8 years^a^** | **PDA Stent (n=26)** | | **BTT Shunt (n=16)** | |
| --- | --- | --- | --- | --- |
|  | **No concern for delay (low risk category)** | **Concern for delay (moderate or high-risk category)** | **No concern for delay (low risk category)** | **Concern for delay (moderate or high-risk category)** |
| **Overall** | **18 (69%)** | **8 (31%)** | **6 (38%)** | **10 (62%)** |
| **Cognitive** | **24 (92%)** | **2 (8%)** | **10 (63%)** | **6 (37%)** |
| **Receptive Language** | **23 (88%)** | **3 (12%)** | **11 (67%)** | **5 (33%)** |
| **Expressive Language** | **23 (88%)** | **3 (12%)** | **9 (56%)** | **7 (44%)** |
| **Fine Motor** | **24 (92%)** | **2 (8%)** | **11 (67%)** | **5 (33%)** |
| **Gross Motor** | **24 (92%)** | **2 (8%)** | **15 (94%)** | **1 (6%)** |
| **Self-Help** | **24 (92%)** | **2 (8%)** | **12 (75%)** | **4 (25%)** |
| **Behavioral** | **22 (85%)** | **4 (15%)** | **14 (88%)** | **2 (12%)** |

**Supplemental Figure S2. Percentages of low-risk category patients (no parental concern for developmental delay) on PEDS Questionnaire for patients ages 2 to 8 years, excluding those with genetic syndromes known to affect neurodevelopment. More children in the PDA stent group were found to fall in the low-risk category for parental neurodevelopmental concerns compared to the BTT shunt group. A statistically significant difference was seen in the cognitive and expressive language domains. Detailed information on age range at time of PEDS questionnaire are available in Supplemental Tables S8 and S9.**


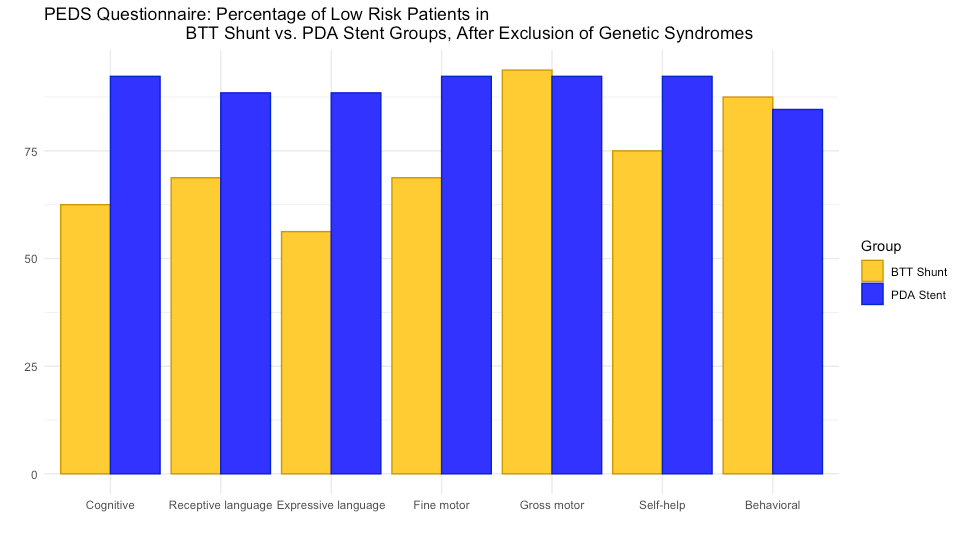


Percent

**Supplemental Table S6. Feeding Modes for Whole Cohort of Patients (n=99) at different time points. There was no significant difference between exclusive PO intake and not exclusive PO intake between the BTTS and PDAS groups at time of shunt or stent nor at time of Glenn or definitive surgery. At time of most recent follow up 79% of patients in the PDAS were exclusively PO feeding versus 68% in the BTTS group. The BTT shunt group was more likely to have ever required a gastrostomy tube for feeds compared to the PDAS group (46% in the BTTS group versus 21% in the PDAS group). PO: per os.**

|  | | **PDAS(n=58)** | **BTTS (n=41)** |
| --- | --- | --- | --- |
| **Mode of Feeding at Time of PDAS or BTTS** | **Exclusive PO**  **Not exclusive PO** | **14 (34%)**  **27 (66%)** | **22 (38%)**  **36 (62%)** |
| **Mode of Feeding at Time of Glenn or Definitive Surgery** | **Exclusive PO**  **Not exclusive PO**  **Unknown** | **22 (54%)**  **15 (36%)**  **4 (10%)** | **31 (53%)**  **9 (16%)**  **18 (31%)** |
| **Mode of Feeding at Most Recent Follow-up** | **Exclusive PO**  **Not exclusive PO**  **Unknown** | **28 (68%)**  **7 (17%)**  **6 (15%)** | **46 (79%)**  **9 (16%)**  **3 (5%)** |
| **Gastrostomy Tube Feedings** | **Yes**  **No**  **Unknown** | **19 (46%)**  **22 (54%)**  **0 (0%)** | **12 (21%)**  **43 (74%)**  **3 (5%)** |

**Supplemental Table S7. Mode of feeding for all patients who received a Bayley-III Assessment and/or PEDS Questionnaire, excluding patients with genetic syndromes known to affect neurodevelopment. There was no significant difference between exclusive oral intake and not exclusive PO intake between the BTT shunt and PDA stent groups at time of shunt or stent, at time of Glenn or definitive surgery, or for mode of feeding at most recent follow-up. However, the BTT shunt group was more likely to have ever required a gastrostomy tube. PO: per os.**

|  | | **PDAS (n=31)** | **BTTS (n=25)** |
| --- | --- | --- | --- |
| **Mode of Feeding at Time of PDAS or BTTS** | **Exclusive PO**  **Not exclusive PO** | **6 (19%)**  **25 (81%)** | **11 (44%)**  **14 (56%)** |
| **Mode of Feeding at Time of Glenn or Definitive Surgery** | **Exclusive PO**  **Not exclusive PO**  **Unknown** | **17 (55%)**  **6 (19%)**  **8 (26%)** | **12 (48%)**  **11 (44%)**  **2 (8%)** |
| **Mode of Feeding at Most Recent Follow-up** | **Exclusive PO**  **Not exclusive PO**  **Unknown** | **26 (84%)**  **5 (16%)**  **0(0%)** | **20 (80%)**  **3 (12%)**  **2 (8%)** |
| **Gastrostomy Tube Feedings** | **Yes**  **No**  **Unknown** | **7 (23%)**  **23 (74%)**  **1 (3%)** | **13 (52%)**  **12 (48%)**  **0(0%)** |

**Supplemental Table S8. Detailed Age Range at time of PEDS Questionnaire for BTTS group**

| **Age at the time of PEDS questionnaire (Table 3)** | **# of Patients** |  | **Age at the time of PEDS questionnaire (Supplemental Table S5)** | **# of Patients** |
| --- | --- | --- | --- | --- |
| **4** | **2** |  | **4** | **1** |
| **5** | **3** |  | **5** | **3** |
| **6** | **7** |  | **6** | **6** |
| **7** | **6** |  | **7** | **4** |
| **8** | **2** |  | **8** | **2** |

**Supplemental Table S9. Detailed Age Range at time of PEDS Questionnaire for PDAS group**

| **Age at the time of PEDS questionnaire (Table 3)** | **# of Patients** |  | **Age at the time of PEDS questionnaire (Supplemental Table S5)** | **# of Patients** |
| --- | --- | --- | --- | --- |
| **2** | **3** |  | **2** | **3** |
| **3** | **4** |  | **3** | **4** |
| **4** | **8** |  | **4** | **7** |
| **5** | **5** |  | **5** | **4** |
| **6** | **4** |  | **6** | **4** |
| **7** | **2** |  | **7** | **1** |
| **8** | **4** |  | **8** | **3** |
